# MXene/Carbon Dots Nanozyme Composites for Glutathione Detection and Tumor Therapy

**DOI:** 10.3390/nano14131090

**Published:** 2024-06-25

**Authors:** Xiaofei Lu, Jingjing Jia, Zonghua Wang, Wenjing Wang

**Affiliations:** College of Chemistry and Chemical Engineering, Shandong Sino-Japanese Center for Collaborative Research of Carbon Nanomaterials, Instrumental Analysis Center of Qingdao University, Qingdao University, Qingdao 266071, China; 2021020368@qdu.edu.cn (X.L.); 2022020328@qdu.edu.cn (J.J.); wangzonghua@qdu.edu.cn (Z.W.)

**Keywords:** carbon dots, MXene, GSH detection, peroxidase-like activity, photothermal therapy

## Abstract

Co-N-CDs-based MXene nanocomposites (MXene@PDA/Co-N-CDs) were constructed by decorating Co-N-CDs on polydopamine-functionalized MXene nanosheets. Both Co-N-CDs and MXene nanosheets have peroxidase-like activity; when the two materials are combined to form MXene@PDA/Co-N-CDs nanocomposites, the peroxide-like activity can be further enhanced. MXene@PDA/Co-N-CDs could oxidize the substrate 3,3′5,5′-tetramethylbenziline (TMB) to form ox-TMB, as confirmed by detecting the absorption of the blue products. A highly selective colorimetric biosensor was developed for the determination of glutathione (GSH) in the concentration range of 0.3 to 20 µM with a lower detection limit (LOD) of 0.12 µM, which realized the accurate detection of GSH in human serum and urine samples. Moreover, in the tumor microenvironment, MXene@PDA/Co-N-CDs could catalyze hydrogen peroxide to produce hydroxyl free radicals and produce a photothermal effect under the exposure of NIR-I irradiation. The catalytic activity of MXene@PDA/Co-N-CD nanocomposites was fully achieved for the death of cancer cells through photothermal/photodynamic synergistic therapy. The MXene@PDA/Co-N-CDs nanozyme offers multiple applications in GSH detection and tumor therapy.

## 1. Introduction

The superior biocompatibility, low cytotoxicity, and good photostability of carbon dots (CDs) position them as highly promising candidates for biomedical applications. Their distinctive characteristics and properties offer exciting opportunities for advancements in biosensing, bioimaging, and nanomedicine [1,2,3]. Besides that, CDs have also been identified as excellent candidates for nanozymes because of their peroxidase (POD)-like activity [4]. Many efforts have been made to improve the catalytic efficiency of carbon dots through surface modification [5,6], heteroatom doping [7], and composite with other nanomaterials [8]. In the application of CDs, nitrogen doping significantly increases their peroxidase activity. As an effective material modification method, nitrogen doping provides strong support for peroxidase activity by increasing the quantity of active sites, thus facilitating the transfer of electrons among substrates [9].

MXene is a transition metal carbide containing either nitrides or carbonitrides with a two-dimensional layered structure. Its general formula is M_n+1_X_n_T_x_, in which M represents the transition metal site [10,11,12,13], X stands for carbon and/or nitrogen, and Tx indicates functional groups on the surface of the outer transition metal layers. It is widely used in catalysis [10,11], energy storage [12], and chemical sensing [13] because it has a high surface area, high catalytic activity, abundant functional groups, and so on [14]. MXene-based nanozymes have aroused significant attention because of their distinctive catalytic and physicochemical properties, offering exciting prospects in the domains of biology and nanomedicine, especially pertaining to medical diagnostics [15,16]. However, MXene tends to aggregate and precipitate in biological media or complex physiological conditions. Surface functionalization of pristine MXene via covalent and noncovalent modifications offers a promising approach to enhance their targeting properties, oxidation/thermal stability, and biocompatibility [17,18].

In addition, MXene has a high spectral absorption rate, excellent photothermal capacity, and specific surface activity, which can absorb NIR-I light, and thus has potential prospects in the fields of biosensing, photoacoustic imaging, and photothermal therapy (PTT) [19]. Ti_3_C_2_-MXene is a rising star among transition metal carbonitride MXenes as a photothermal agent with prominent photothermal conversion and thermal conductivity. However, the further practical applications of MXene may be limited by some factors, such as its photothermal capacity, peroxidase-like activity, sensitivity, and selectivity [14,20]. So, it is of vital importance to fabricate MXene with other nanomaterials to achieve successful cancer therapy or catalytic activity. For example, Zhu et al. [15] developed a Ti_3_C_2_-based nanocomposite (Ti_3_C_2_Tx-Pt-PEG) to realize the catalytic activity of hyperthermia-amplified nanoenzymes for imaging-guided synergistic cancer therapy.

Glutathione (GSH), an important tripeptide molecule, is composed of three amino acid units including glutamic acid, cysteine, and glycine [21]. Being an efficient antioxidant and detoxifier, it plays a pivotal role in maintaining cellular redox balance. At present, although the importance of GSH in biology and medicine has been widely recognized, effective GSH detection methods are still being explored. For the prompt and precise diagnosis and treatment of significant illnesses including diabetes, Alzheimer’s disease, and cancer [22,23], it is of utmost importance to establish a dependable and accurate GSH detection method. There are many methods for GSH detection, such as capillary electrophoresis [24], electrochemistry [25], MS surface-enhanced Raman scattering [26], HPLC [27], etc. Although a variety of techniques have emerged in the field of GSH detection, these highly sensitive techniques are often accompanied by long pretreatment times, difficult operation, and high cost, making it impossible to detect quickly clinically [28]. With the progress of technology and the improvement in methods, fluorescence and colorimetry have gained broad application prospects as simple, rapid, and sensitive GSH detection methods.

**Scheme 1:**
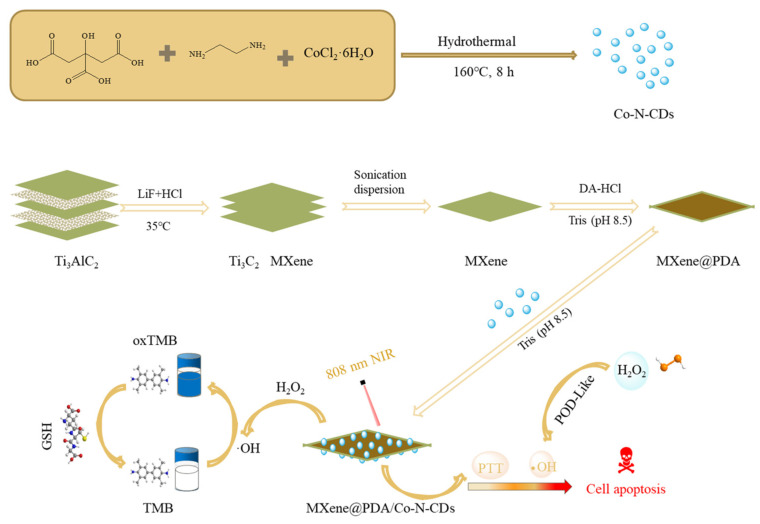
Illustrative representation of the synthesis process for MXene@PDA/Co-N-CDs nanocomposites and the applications in colorimetric detection of GSH and hyperthermal-enhanced catalytic therapy of tumors.

In this study, Co and N co-doped CDs (Co-N-CDs) are synthesized by employing citric acid, ethylenediamine, and CoCl_2_ as precursors. Polydopamine (PDA) has the advantage of biocompatibility, excellent hydrophilicity, and ease of modification with other materials. Here, with the coating of PDA, the introduction of MXene@PDA significantly improves the dispersion and electron transport ability of nanocomposites and provides a deposition matrix. Leveraging the synergistic interaction between MXene and Co-N-CDs, in the catalytic process, MXene@PDA/Co-N-CDs can promote the conversion of oxygen molecules into hydroxyl radicals and then trigger the oxidation reaction of TMB. The Michaelis constant is as low as 0.017 mM. The composite has abundant active sites and excellent catalytic performance of peroxidase activity. Utilizing the combined effects of MXene and Co-N-CDs, it serves as a highly sensitive and selective colorimetric sensing platform for GSH detection, achieving a limit of detection (LOD) of 0.12 µM. More importantly, the superthermal enhanced catalytic therapeutic properties of MXene@PDA/Co-N-CDs under NIR laser light irradiation have opened a new and promising method for the catalytic treatment of tumors mediated by nanozymes (Scheme 1).

## 2. Materials and Methods

### 2.1. Materials and Apparatuses

Citric acid (CA), CoCl_2_·6H_2_O, HCl, H_2_O_2_, Acetic acid, Sodium acetate, glycine, KCl, MgCl_2_, NaCl, FeCl_3_, KI, ZnCl_2_, Ca(OH)_2_, urea, glucose, and Ethanol were obtained from Sinopharm Chemical Reagents Co., Ltd. (Shanghai, China) 3,3′,5,5′-tetramethyl benzidin (TMB), EDA·H_2_O, Ti_3_AlC_2_, LiF, dopamine hydrochloride (DA-HCl), methylene blue (MB), and Chitosan were obtained from Aladdin (Shanghai, China). Hydroxymethyl (Tris), glutathione (GSH), L-Glutamic acid, L-Cysteine, L-isoleucine, and Sodium citrate were obtained from Macklin (Shanghai, China). PBS, DMEM high glucose, and fetal bovine serum were obtained from Solarbio (Beijing, China). Pancreatin was obtained from Gibco (Shanghai, China). CCK-8, DCFH-DA, and crystal violet staining solution were obtained from Beyotime Biotechnology (Shanghai, China). The Annexin V-FITC/PI apoptosis detection kit was purchased from Meilun Biotechnology Co., Ltd. (Dalian, China).

Transmission electron microscopy (TEM) was performed with HT7700 (Hitachi, Tokyo, Japan), and high-resolution TEM (HRTEM) was performed with JEOLJEM 2100F (JEOL, Tokyo, Japan). The elemental compositions were measured with photoelectron spectroscopy (XPS) (D8 Advance, Bruker, Germany). The X-ray diffraction (XRD) spectra were obtained with Smart Lab 3KW at room temperature (Rigaku Corporation, Tokyo, Japan). Atomic force microscopy (AFM) was performed on Dimension Icon (Bruker, Billerica, MA, USA). The UV-vis spectra were obtained by using a UV-2700 UV-vis spectrophotometer (Rigaku Corporation, Japan). Fluorescence spectra were collected on a fluorescent spectrometer (F-7000, Hitachi Ltd., Japan). The spectrophotometer was obtained by using a SpectraMax i3x (Molecular Devices, Urstein, Austria).

### 2.2. Synthesis of MXene

During the experiment, 0.8 g LiF was added to a 10 mL HCl (9 M) PTFE reaction kettle, stirring for 5 min to ensure that it was fully dissolved. Then, 0.5 g Ti_3_AlC_2_ was slowly added to the previously prepared solution and continuously stirred at 3 °C for 24 h to obtain an adequate reaction. Subsequently, the obtained mixture was rinsed a few times with distilled water, centrifuged at 3500 rpm through a centrifuge, and the process was repeated many times until the pH of the supernatant attained a value of 6. Then, water was added to the solid precipitate to 100 mL followed by ultrasonic treatment for 1 h under nitrogen protection. The mixture was centrifuged again at 3000 rpm for 1 h. The supernatant was subjected to freeze-drying within the centrifugal tube to facilitate its subsequent utilization.

### 2.3. Preparation of MXene@PDA

First, 100 mg of MXene was dispersed in 100 mL of Tris-HCl buffer solution, then 100 mg of dopamine hydrochloride was added into the mixture followed by stirring in the dark for 24 h at 45 °C. Finally, in order to obtain dry MXene@PDA, the MXene@PDA precipitate obtained by centrifugation was placed in a freeze dryer, and the moisture in the sample was completely removed by low-temperature freezing and vacuum drying; thus, the dry MXene@PDA powder was obtained.

### 2.4. Synthesis of Co-N-CDs

Here, a hydrothermal technique was employed for the synthesis of Co-N-CDs. First, 2.0 g CA was dissolved in 20 mL distilled water. Then, 0.04 g CoCl_2_·6H_2_O was added, and the mixture was continuously stirred for five minutes until fully dissolved. Then, 85 μL EDA·H_2_O was added to the mixture. After mixing, the resulting mixture was carefully transferred to the PTFE reactor, and the temperature was kept stable at around 160 °C to provide a stable reaction environment. After 8 h of the heating reaction, we obtained the initial product. After the high-temperature reaction, we remove the reactor and allow it to cool naturally to room temperature. Once the product reached room temperature, the mixture was filtered using a 0.22 μm membrane filter (aquo-system) to remove large impurity particles from the mixture and ensure the purity of the product. The filtered solution then underwent dialysis for 48 h to remove unreacted small molecules and further improve the purity of the product. Finally, we freeze-dried the solution after dialysis and successfully obtained a pure and dry product.

### 2.5. Synthetic MXene@PDA/Co-N-CDs

First, 100 mg MXene@PDA and Co-N-CDs were precisely weighed and carefully dispersed in 100 mL of Tris-HCl buffer solution, and then the mixture was agitated at 45 °C for a duration of 24 h to ensure thorough mixing and reaction. After completing the above steps, we centrifuged and washed the mixture. Finally, through freeze-drying technology, we successfully obtained products ready for further use. The schematic fabrication process of MXene@PDA/Co-N-CDs nanocomposites is shown in Scheme 1.

### 2.6. Exploration of the POD-Like Activity of MXene@PDA/Co-N-CDs

Exploiting the catalytic oxidation capacity of TMB in the presence of hydrogen peroxide, we successfully evaluated the peroxide-like activity of MXene@PDA/ Co-N-CDs. MXene@PDA/Co-N-CDs (10 μg·mL^−1^), hydrogen peroxide (50 mM), TMB (5 mM), and NaAC-HAC (pH 3.6) were mixed into a 2 mL centrifuge tube and reacted in a water bath at 40 °C for 12 h. The absorbance at 652 nm was recorded. The impact of pH (from 3.0 to 5.0) and temperature (from 25 °C to 55 °C) were determined to investigate their effects on the POD-like activity of MXene@PDA/Co-N-CDs.

### 2.7. ·OH Monitored by Using MB

First, 10 μg·mL^−1^ of the MXene@PDA/Co-N-CDs nanocomposites was added to a freshly prepared methylene blue solution (MB, H_2_O, 2 μg·mL^−1^) containing 50 mM hydrogen peroxide. After incubation at different time intervals, the absorbance of λ = 664 nm was determined on a UV–visible spectrophotometer.

### 2.8. Steady-State Kinetic Analysis of MXene@PDA/Co-N-CDs

The steady-state kinetics of the MXene@PDA/Co-N-CDs nanocomposites (10 μg·mL^−1^) in the presence of NaAc-HAc (pH 3.6) were investigated. Different concentrations of TMB solution or hydrogen peroxide were added, and the absorbance at 652 nm of the solution was then determined. After further analysis of the experimental data, the Michaelis–Menten saturation curve was obtained. Finally, the Michaelis constant of MXene@PDA/Co-N-CDs when used as peroxidase was obtained through calculation.

### 2.9. H_2_O_2_ Detection

To explore the catalytic process of MXene@PDA/Co-N-CDs as a peroxide mimic enzyme and substrate, 200 μL TMB (5 mM), 300 μL NaAC-HAC (pH 3.6), 500 μL of various concentrations of hydrogen peroxide, and 500 μL MXene@PDA/Co-N-CDs (10 μg·mL^−1^) were added to a 2 mL centrifuge tube. After mixing thoroughly and incubating for 12 h at room temperature, a UV-vis spectrophotometer was used to measure the absorbance at λ = 652 nm.

### 2.10. Detection of GSH

First, 200 μL TMB (5 mM), 300 μL NaAC-HAC buffer solution (pH 3.6), MXene@PDA/Co-N-CDs (10 μg·mL^−1^ 500 μL), and 50 mM H_2_O_2_ (500 μL) solution were successively added to 2 mL centrifuge tubes and incubated for 12 h. Then, 500 μL GSH solution in a series of concentration gradients was added to the mixture and incubated at normal temperature for 20 min to ensure that glutathione fully reacted with MXene@PDA/Co-N-CDs. Finally, a UV-vis spectrophotometer was utilized to measure the absorbance at λ = 652 nm accurately.

### 2.11. Detection of Glutathione in Human Urine and Serum Samples

To verify the practicality and applicability of our proposed method for detecting catalytic activity based on MXene@PDA/Co-N-CDs in actual sample detection, we further extended our investigation to the detection of GSH in human urine and serum samples. In this study, we used morning urine provided by 3 volunteers as experimental samples. The urine samples were pretreated, and the solid particles and impurities were removed by centrifugation (1200 rpm, 40 min) to ensure the accuracy and reliability of subsequent analysis. Subsequently, we diluted the urine sample 10-fold with HAC-NaAC solution (pH 3.6) for further use. Serum samples were provided by the Affiliated Hospital of Qingdao University. The experiments on detecting GSH in human serum complied with relevant laws and institutional guidelines and were approved by Qingdao University. The serum samples were centrifuged (12,000 rpm, 5 min), and the supernatant was diluted 50-fold with HAC-NaAC solution (pH 3.6).

A total of 200 μL 5 mM TMB, 300 μL NaAC-HAC buffer solution (pH 3.6), 500 μL 10 μg·mL^−1^ Co-N-CDs, and 500 μL 50 mM of H_2_O_2_ solution were added to 2 mL centrifuge tubes successively. After 12 h of incubation, the diluted real samples and different spiked concentrations of GSH were added into the above tubes for recovery experiments. We then incubated the mixture at normal temperature for 20 min. After incubation, the absorbance at λ = 652 nm was accurately measured.

### 2.12. Photothermic Performance of MXene@PDA/Co-N-CDs In Vitro

The temperature changes and thermal images of Co-N-CDs, MXene, and MXene@PDA/Co-N-CDs nanocomposites at different times were measured by using an infrared thermal camera. We used an 808 nm laser light (2 W·cm^−2^) to irradiate the samples at normal temperature for 10 min. At the same time, in order to more fully evaluate the impact of laser light irradiation on the properties of the material, we also irradiated purified water under the same conditions.

### 2.13. Cytotoxicity Test by the CCK-8 Assay

The cytotoxicity of MXene@PDA/Co-N-CDs was tested in human breast cancer cells (MCF-7). MCF-7 cells were seeded on a 96-well plate and cultured in the incubator for 24 h. Then, different concentrations of MXene@PDA/Co-N-CDs (0, 100, 250, 500, 750, 1000 μg·mL^−1^) and DMEM high glucose medium were added and incubated for 4 h. For comparison, the light group was illuminated with 808 nm laser light (2 W·cm^−2^) for 10 min, and the medium without MXene@PDA/Co-N-CDs was the blank group. Next, 100 μL 10% CCK-8 was added to all the wells. After incubation in the incubator for 0.5–1 h, the absorbance was measured in a microplate reader, and then the level of cytotoxicity was determined.

### 2.14. Apoptosis Detection

MCF-7 cells were inoculated into Petri dishes and further incubated in an incubator. Once the cells reached the desired state, the original culture medium was removed and replaced with culture medium containing MXene@PDA/Co-N-CDs (500 µg·mL^−1^) or substituted with fresh culture medium, following an additional incubation for 4 h. Subsequently, the cells were digested, centrifuged (1000 rpm, 3 min), washed twice with PBS, redispersed in buffer solution, and stained with Annexin-FITC/PI for 15 min. Finally, the extent of apoptosis was detected using a flow cytometer.

### 2.15. Clonogenic Assay

Initially, MCF-7 cancer cells were seeded at a density of 2000 cells per well for 24 h, followed by the addition of MXene@PDA/Co-N-CDs nanocomposites and incubation for another 4 h. Subsequently, the laser light group was exposed to 808 nm near-infrared laser irradiation. After 8 days of culture, the cells were fixed with paraformaldehyde for 15 min. The plate was then cleaned three times with deionized water and 1 mL crystal violet dye was added for 20 min of dyeing. Then, the plate was washed again, air-dried, and photographed. Finally, the clones from all groups were counted, and the survival rate was plotted.

### 2.16. Intracellular ROS Detection

The intracellular ROS levels of the MXene@PDA/Co-N-CDs nanocomposites were measured by 2,7-dichlorofluorescein diacetate (DCFH-DA). First, MCF-7 cells were seeded in confocal plates; then, the cells were mounted with different concentrations of MXene@PDA/Co-N-CDs and cultured for an additional 4 h. Then, another confocal plate was incubated with 500 μg·mL^−1^ MXene@PDA/Co-N-CDs for 4 h. Then, 1 mL DCFH-DA (0.01 mM) was added to all confocal plates and incubated for 20 min. After that, they were irradiated with 808 nm laser light at 2 W·cm^−2^ for 10 min. Finally, the cells were washed with PBS, and cellular ROS signals were visualized by a fluorescence-inverted microscope. The intracellular ROS levels of MCF-7 were measured by the following treatments: (1) control, (2) laser light, (3) MXene@PDA/Co-N-CDs (500 μg·mL^−1^), and (4) MXene@PDA/Co-N-CDs (500 μg·mL^−1^) under laser light.

## 3. Results and Discussion

### 3.1. Characterization

**Figure 1:**
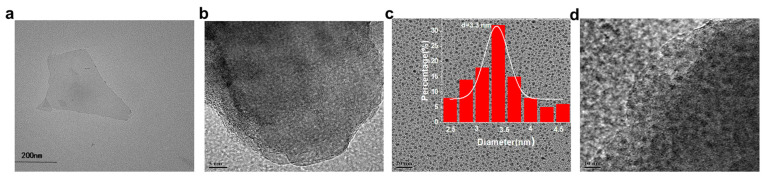
TEM images of MXene (**a**), MXene@PDA (**b**), Co-N-CDs (**c**), and MXene@PDA/Co-N-CDs (**d**).

Transmission electron microscopy (TEM) provided a comprehensive visualization of the morphologies of Co-N-CDs, MXene, MXene@PDA, and MXene@PDA/Co-N-CDs separately. By reaction in a mixed fluoride salt/hydrochloric acid system, the Al layer in Ti_3_AlC_2_ was successfully etched to obtain an MXene nanosheet with a smooth surface with a single layer (Figure 1a) [29].

Figure 1b shows that a light-colored boundary layer was clearly visible along the edges of MXene, unequivocally indicating the successful coating of PDA on the surface of MXene [30]. The spherical Co-N-CDs in Figure 1c are uniformly monodisperse, with an average diameter of approximately 3.3 nm. Furthermore, Figure 1d clearly shows that the carbon dots bind tightly on the MXene@PDA surface to form the MXene@PDA/Co-N-CDs. These results demonstrate that MXene@PDA/Co-N-CDs composites were successfully prepared.

Based on the AFM image (Figure S1a) analysis, the thickness of MXene nanosheets obtained was relatively uniform, and the average height of MXene was 1.02 nm, indicating that monolayer MXene nanosheets were successfully obtained.

Using a Zeta analyzer, the potential of MXene was measured to be −27.3 mV. Because of the presence of a large number of −OH, −O, and −F groups, MXene exhibits a high electronegativity [10]. After coating PDA on MXene, the Zeta potential was obviously decreased to −44.9 mV because PDA contains more electronegativity groups, such as −OH and −NH groups. The Zeta potential of CDs was measured at −0.3 mV. Finally, with the loading of MXene@PDA, the potential of MXene@PDA/Co-N-CDs was −21.7 mV, further indicating the successful preparation of MXene/CD-based nanocomposites (Figure S1c).

**Figure 2:**
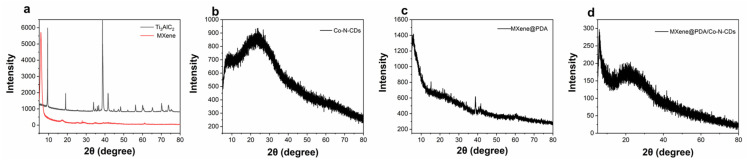
XRD patterns of MAX, MXene (**a**), MXene@PDA (**b**), Co-N-CDs (**c**), and MXene@PDA/Co-N-CDs (**d**).

XRD was used to confirm the structure of the MXene nanosheet. Compared with Ti_3_AlC_2_, the MAX phase of the MXene nanosheet of the (002) peak shifted from 9.5° to 5.91°. The disappearance of the peaks at 39.5° indicated that the Al layer was completely etched away [31,32] and the MXene nanosheet was successfully obtained (Figure 2a). As shown in Figure 2b, compared with pure MXene, the peak value of MXene coated with PDA was reduced to 5.57°, suggesting that the addition of the PDA coating may lead to an increase in interlayer spacing.

**Figure 3:**
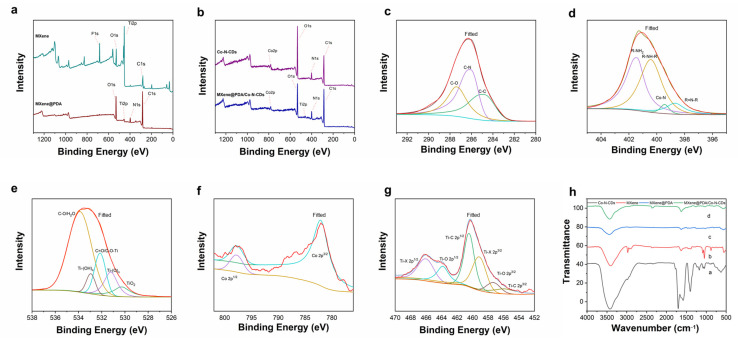
XPS patterns of MXene and MXene@PDA (**a**), Co-N-CDs and MXene@PDA/ Co-N-CDs (**b**). XPS high-resolution scan of C 1s (**c**), N 1s (**d**), O1s (**e**), Ti 2p (**f**), and Co 2p (**g**) in MXene@PDA/Co-N-CDs. FT-IR spectra of Co-N-CDs, MXene, MXene@PDA, and MXene@PDA/Co-N-CDs (**h**).

Figure 3a,b show the full spectrum XPS data for MXene, MXene@PDA, Co-N-CDs, and MXene@PDA/Co-N-CDs. Figure 3a shows the XPS measurement spectra of MXene and MXene@PDA. These spectral data clearly confirm the presence of C, O, F, and Ti elements. Furthermore, the N element was found in the spectrum of MXene@PDA. MXene@PDA/Co-N-CDs revealed the presence of the C, N, Ti, Co, and O elements. The C1s XPS spectrum of MXene@PDA/Co-N-CDs was divided into three peaks (Figure 3c), corresponding to C-C (284.28 eV), C-O-Ti (287.4 eV), and C-O/C-N (285.94 eV). The XPS spectra of N 1s (Figure 3d) were consistent with four components, including a primary amine (R=N-R) at 398.7 eV, a tertiary amine (R-NH-R) at 400.45 eV, a primary amine (R-NH_2_) at 401.05 eV, and Co-N at 399.45 eV [29,33]. The O 1s region (Figure 3e) had five characteristic peaks at 530.3, 531.25, 532.15, 532.95, and 533.9 eV, which were ascribed to titanium dioxide, Ti-(O)x, C=O/C-O-Ti, Ti-(OH)x, and C-O/H_2_O, respectively [34]. The Ti 2p spectrum (Figure 3f) was divided into three pairs of spin peaks of Ti 2p^3/2^ and Ti 2p^1/2^. The four functional components that appeared at 455.75, 460.5, 466.15, and 459.2 eV were correlated with Ti-C 2p^3/2^, Ti-C 2p^1/2^, Ti-X 2p^1/2^, and Ti-X 2p^3/2^, respectively. As evident in Figure 3g, the Co 2p energy spectrum showed significant peaks of Co 2p^3/2^ and Co 2p^1/2^ at 781.38 and 797.21 eV, respectively. The interval between these two peaks was 15.83 eV, indicating the presence of Co(III). In addition, the presence of Co(II) was confirmed by the XPS spectra of the Co 2p^3/2^ peak and the recombinant satellite peak at 786.94 eV, providing the structural foundation for the enzymatic reaction [9].

The FT-IR spectra of MXene, MXene@PDA, Co-N-CDs, and MXene@PDA/Co-N-CDs are shown in Figure 3h. The characteristic absorption bands at 3425, 1779, 1706, 1572, 1173, and 1235 cm^−1^ belonged to NH/N-H, C=O, C=N, C=C, C-N, and C-O vibrations of Co-N-CDs, respectively, which indicated that the surface contained many hydrophilic groups such as amino and hydroxyl groups. In addition, the peak at 624 cm^−1^ was attributed to the tensile vibration of Co−N, and this indicated that cobalt doping was successful and provided active sites for nanozymes [9]. The peak at 3418 cm^−1^ was the tensile vibration of -OH in MXene (curve b), while the tensile vibrations of C=O, C–O, and C-F were located at 1630 cm^−1^, 1091 cm^−1^, and 1049 cm^−1^, respectively. The peak value at 558 cm^−1^ was the characteristic peak of the Ti-O bond [30].

For MXene@PDA (curve c), two intense characteristic peaks at 3419 and 1618 cm^−1^ were observed in the FT-IR spectrum. According to previous literature reports [35], these two characteristic peaks correspond to the aromatic ring structure and the catechole–OH group in the PDA molecule, respectively. More abundant characteristic peaks were observed in the FT-IR spectra (curve d) of MXene@PDA/Co-N-CDs composites. These characteristic peaks included both the strong absorption peak of MXene@PDA and the characteristic absorption peak of Co-N-CDs. The results fully show that Co-N-CDs were successfully introduced into the MXene@PDA system to form MXene@PDA/Co-N-CDs.

### 3.2. Peroxidase-like Activity of MXene@PDA/Co-N-CDs

Co-N-CDs and MXene both showed significant peroxisase-like activity. Specifically, when the two nanomaterials reacted with the TMB substrate in the presence of hydrogen peroxide, they were able to catalyze the oxidation of the TMB substrate. Figure S2 explores in detail how three key factors, i.e., pH, temperature, and TMB concentration, affect the catalytic activity of MXene@PDA/Co-N-CDs composite. The results showed that MXene@PDA/Co-N-CDs had good enzymatic activity at a temperature of 40 °C, the TMB concentration was 5 mM, and the pH value was 3.6.

**Figure 4:**
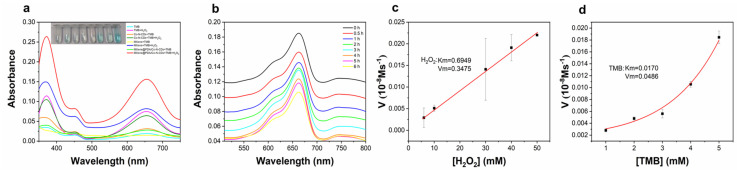
UV–vis absorption spectra of different catalytic systems (**a**); UV-vis absorbance spectra of MB (1 mg L^−1^) as substrate catalyzed by MXene@PDA/Co-N-CDs in the presence of H_2_O_2_ (50 mM) (**b**) and steady-state kinetic analysis of MXene@PDA/CDs (**c**,**d**).

The UV absorption spectra in the different systems are shown in Figure 4a. After mixed reactions in different systems at the optimal temperature and pH, it was obvious that the UV–visible absorbance of MXene@PDA/Co-N-CDs was higher than the other substances, indicating a higher peroxidase-like activity.

The catalytic pathways for peroxidase-like activity usually involve two core processes including reactive oxygen species generation and electron transfer processes that occur during this reaction. In the current study system, in order to reveal potentially active intermediates, we used methylene blue (MB) as a probe to monitor the formation of hydroxyl (·OH) radicals [36]. The hydroxyl radical, as a highly active oxidant, can react with MB, resulting in a significant decrease in the absorbance intensity of MB. As shown in Figure 4b, we observed the absorbance changes in solutions containing MB, hydrogen peroxide, and MXene@PDA/Co-N-CDs during the reaction. After a reaction period of 6 h, it became evident that the absorbance of the solution underwent a notable decrease. This experimental phenomenon strongly proves the existence of ·OH free radicals and further confirms that CD composites can produce ·OH free radicals during the catalytic process, thus exhibiting peroxidase activity. CDs activated the decomposition process of hydrogen peroxide, thus promoting the oxidation of TMB. With the addition of GSH, the dark blue hue of oxTMB gradually faded away. The corresponding UV-vis spectra showed that the absorption peak near 652 nm was significantly reduced, indicating that the oxTMB could be reduced by GSH.

To investigate the POD simulation activity of MXene@PDA/Co-N-CDs systematically, we explored the steady-state kinetics at different concentrations of hydrogen peroxide and TMB under the optimal conditions described above. The results showed that both hydrogen peroxide and TMB conform to the standard Michahelis–Menten model (Figure 4c,d). By fitting with the Lineweaver Burk plot as 1/V = (Km/Vmax) (1/[S]) + 1/Vmax, the maximum initial velocity (Vmax) and Miter constant (Km) corresponding to hydrogen peroxide and TMB were obtained, as shown in Table 1. In addition, compared with HRP and the other related nanozymes reported, MXene@PDA/Co-N-CDs had the minimum Km. Km is a key parameter of enzyme kinetics that reflects the ability of an enzyme to bind to a specific substrate. A low Km value signifies that the enzyme exhibits a stronger affinity for the substrate, subsequently indicating a higher catalytic efficiency towards the substrate. In this study, we observed that the Km value between MXene@PDA/Co-N-CDs and TMB is low. This finding not only confirmed the strong affinity between MXene@PDA/Co-N-CDs and TMB but also revealed its excellent POD-like activity.

### 3.3. Colorimetric Detection of H_2_O_2_ and GSH

To improve the catalytic activity of MXene@PDA/Co-N-CDs towards H_2_O_2_ and GSH, after optimizing the main parameters, a temperature of 40 °C, pH of 3.6, and TMB concentration of 5 mM were selected as the optimum experimental conditions for detecting H_2_O_2_ and GSH (Figure S2).

**Figure 5:**
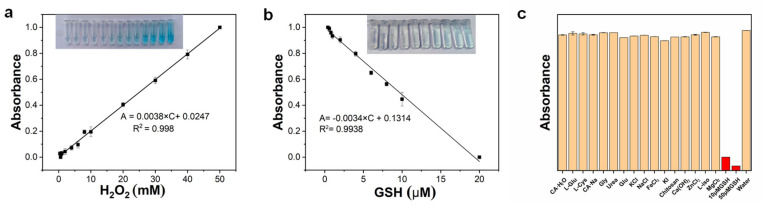
The UV−vis absorption variation photo of MXene@PDA/Co-N-CDs with different concentrations of H_2_O_2_ and its calibrations curve toward 0.4−50 mM (**a**). The correlation between absorbance and GSH concentration with a photo of the corresponding solution (**b**); and the selectivity of MXene@PDA/Co-N-CDs for GSH detection (**c**).

As the concentration of H_2_O_2_ rises, a pronounced shift occurs in the color of the mixture, transitioning smoothly from its initial colorless state to a vibrant blue. Therefore, we performed the colorimetric detection of H_2_O_2_ by observing the color change. Furthermore, we further investigated the relationship between the H_2_O_2_ concentration and absorbance. The experimental results showed that in the concentration range of 0.4 to 50 mM, the concentration of H_2_O_2_ showed a linear correlation with the absorbance at 652 nm (as shown in Figure 5a). The linear regression equation is A = 0.0038 × C + 0.0247, R^2^ = 0.998, with the detection limit of 0.23 mM.

Upon the addition of GSH, the absorbance of the oxTMB-containing solution system gradually decreased at 652 nm. This occurred because the oxidation process of TMB catalyzed by MXene@PDA/Co-N-CDs was inhibited by the addition of GSH because of the competitive effect. As shown in Figure 5b, the absorbance of the reaction system with TMB was significantly proportional to the concentration of GSH in the concentration range of 0.3–20 μM. In this concentration range, the absorbance of the reaction system also increased with the increase in the GSH concentration. It is worth noting that the detection limit of GSH in this method reached 0.12 μM; this finding was comparable to other previously reported works. (Table 2).

The selectivity of the MXene@PDA/Co-N-CDs system for GSH detection was also investigated. Common metal ions, inorganic cations, and other common biological substances were tested including K^+^, Na^+^, Zn^2+^, Mg^2+^, Cl^−^, I^−^, urea, cysteine, glycine, etc. It can be seen in Figure 5c that the selected interfering substance does not affect the catalytic reaction. Even if the concentration of interfering substances introduced in the experimental system was ten times the concentration of glutathione, it would not affect the catalytic reaction, suggesting that MXene@PDA/Co-N-CDs showed good selectivity and specificity for GSH detection.

To validate the utility of this method, it was used to detect GSH in human urine (Table 3) and serum samples (Table 4). The GSH concentrations of diluted serum samples were measured to be 0.178, 0.179, and 0.25 µM, which were equivalent to 27.3 mg/100 g, 27.5 mg/100 g, and 38.4 mg/100 g in the original serum samples. These results were consistent with the report results. To further confirm the accuracy of the method, spiked recovery experiments were carried out. The spiked recoveries were between 96% and 110%, which demonstrated this proposed assay system could become an effective method for GSH determination in real samples.

### 3.4. In Vitro Photothermal Properties of MXene@PDA/Co-N-CDs

**Figure 6:**
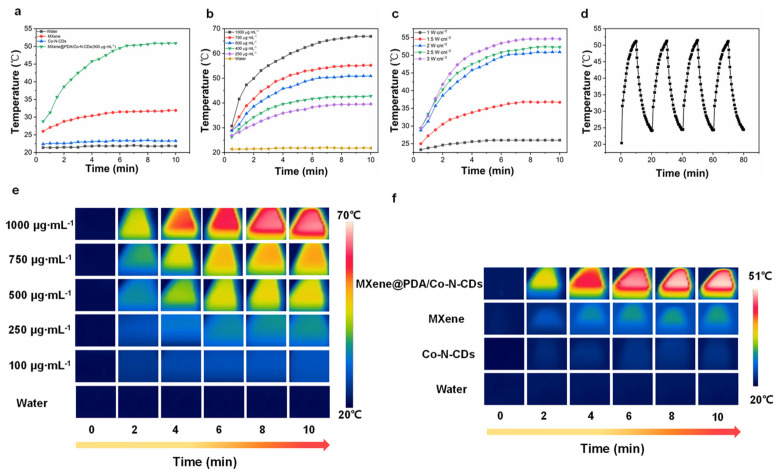
Temperature change curves of various samples (**a**). Temperature change curves of water and MXene@PDA/Co-N-CDs with different concentrations as a function of irradiation time (**b**). NIR irradiation with different power densities (the irradiation wavelength was 808 nm (**c**). Photostability of MXene@PDA/CDs under 808 nm laser light irradiation for four cycles (2 W·cm^−2^) (**d**). Infrared thermal images of pure water and MXene@PDA/Co-N-CDs nanocomposites with various concentrations irradiated with 808 nm laser light (2 W·cm^−2^) for 10 min (**e**). Infrared thermal images of water, MXene, Co-N-CDs, and MXene@PDA/Co-N-CDs nanocomposite aqueous solutions with the same concentration irradiated with 808 nm laser light (2 W·cm^−2^) for 10 min (**f**).

Figure 6a,e show that the hybrid materials combined with MXene and Co-N-CDs exhibited more excellent photothermal properties than MXene and Co-N-CDs themselves. The photothermal conversion performance of MXene@PDA/Co-N-CDs was evaluated by recording the temperature variations and infrared thermography images while irradiating with various concentrations (250 μg·mL^−1^, 400 μg·mL^−1^, 500 μg·mL^−1^, 750 μg·mL^−1^, 1000 μg·mL^−1^, and water) under an 808 nm NIR laser irradiation at 2 W·cm^−2^. As the concentration increased, the temperature elevated gradually. The temperature could be increased by 67 °C for 10 min with 1000 μg·mL^−1^ MXene@PDA/Co-N-CDs compared with pure water. So, the optimal concentration of MXene@PDA/Co-N-CDs was chosen at 500 μg·mL^−1^.

As shown in Figure 6c, the photothermal performance at different power densities (1 W·cm^−2^, 1.5 W·cm^−2^, 2 W·cm^−2^, 2.5 W·cm^−2^ and 3 W·cm^−2^) was also evaluated by keeping the concentration of MXene@PDA/Co-N-CDs at 500.0 μg·mL^−1^. With the increase in laser light power density, a more significant temperature elevation was observed, exhibiting a photothermal effect dependent on the power density.

At the same time, as shown in Figure 6f, laser switching experiments were used to study the photothermal stability of MXene@PDA/Co-N-CDs nanocomposites. In four consecutive heating and cooling cycles, the temperature variations remained nearly identical, indicating its ideal photothermal stability. The high stability of MXene @PDA/Co-N-CDs nanocomposites as a durable photothermal agent was highlighted [15].

### 3.5. Anticancer Cell Effects In Vitro

**Figure 7:**
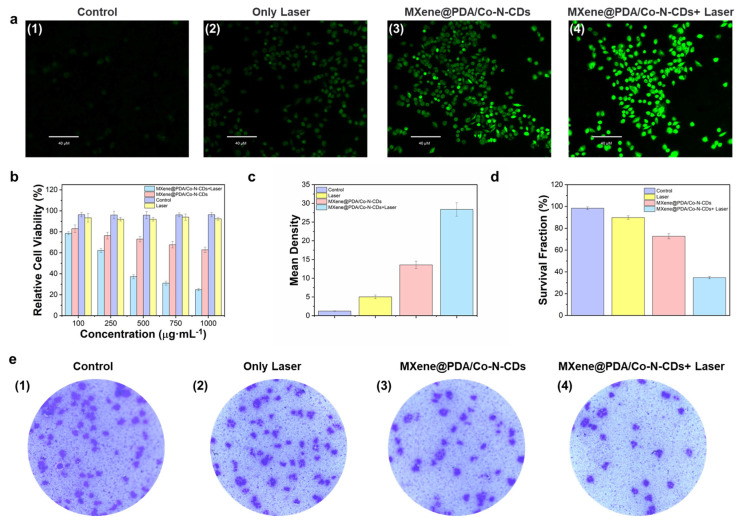
Fluorescence microscope images of the intracellular ROS level of MCF-7 cells with different treatments (the scale bar represents 40 μm): (1) control; (2) 808 nm laser light (2 W·cm^−2^); (3) MXene@PDA/Co-N-CDs only at the concentration of 500 μg·mL^−1^; and (4) MXene@PDA/Co-N-CDs (500 μg·mL^−1^) + 808 nm laser light (2 W·cm^−2^) (**a**). Toxic effects of different formulations on MCF-7 cells by CCK-8. Data are presented as mean ± SD (*n* = 3) (**b**) and corresponding mean fluorescence intensity of intracellular ROS level with different treatments (**c**). The survival fraction (**d**) and images (**e**) of the clonogenic assay with MCF-7 cells treated with different treatments: (1) control; (2) 808 nm laser light (2 W·cm^−2^); (3) MXene@PDA/Co-N-CDs only at the concentration of 500 μg·mL^−1^; and (4) MXene@PDA/Co-N-CDs (500 μg·mL^−1^) + 808 nm laser light (2 W·cm^−2^).

Because of the high POD-like activity of MXene@PDA/Co-N-CDs nanozyme, catalytic therapy in vitro was investigated. ROS production in MCF-7 cells was evaluated by using DCFH-DA as a fluorescent probe. As can be seen in Figure 7a, cells incubated with MXene@PDA/Co-N-CDs exhibited stronger green fluorescence than those in the blank control groups (water and NIR laser light only), indicating that MXene@PDA/Co-N-CDs nanocomposites could effectively catalyze the generation of intracellular ·OH. Moreover, the fluorescence signal was stronger in the NIR laser-irradiated group compared with the MXene@PDA/Co-N-CDs group at the concentration of 500 μg·mL^−1^, showing that the photothermal effect of MXene@PDA/Co-N-CDs could enhance its POD-like catalytic activity.

Cytotoxicity evaluation is a key step in the biocompatibility study of MXene@PDA/Co-N-CDs. The cytotoxicity of MXene@PDA/Co-N-CDs to MCF-7 cells was successfully evaluated by the CCK-8 assay. As shown in Figure 7b, the cell viability treated with laser light was like that of the blank control group, and a slight decrease could be observed in these control groups. MXene@PDA/Co-N-CDs showed a concentration-dependent phenomenon on the cell viability of MCF-7. The cell viability was 83% after cultivation with 100 μg·mL^−1^ MXene@PDA/Co-N-CDs, indicating the biocompatibility of this material. However, it decreased to 72% after cultivation with 500 μg·mL^−1^ MXene@PDA/Co-N-CDs, indicating that ROS were produced at this concentration. In addition, after incubation with 500 μg·mL^−1^ MXene@PDA/Co-N-CDs, the MCF-7 cells treated with laser radiation showed a higher killing effect, and the cell viability of MCF-7 further decreased to 37%. Laser light irradiation improved cell death. Figure 7c also shows that under 808 nm laser irradiation, MXene@PDA/Co-N-CDs produced more intracellular ROS and induced cell apoptosis.

At the same time, the photokilling ability of the MXene@PDA/Co-N-CDs complex on cells was evaluated by the clonogenic assay to record both early and late cell death [48]. As depicted in Figure 7, the colony formation rates of MCF-7 cells treated with 808 nm laser light irradiation and the MXene@PDA/Co-N-CDs nanocomposite were 89% and 72%, respectively. Further data from the clone survival assay indicated that the combination treatment group using MXene@PDA/Co-N-CDs and laser irradiation (MXene@PDA/Co-N-CDs + Laser light) significantly enhanced the therapeutic effect, resulting in a decrease to 34% in the colony formation rate of MCF-7 cells, which may be due to the fact that MXene@PDA/Co-N-CDs nanocomposites produced a large amount of ·OH in cells, which induced the cell death of MCF-7 cells. Additionally, local hyperthermia accelerated the generation of ·OH and enhanced the cell death of MCF-7 cells, further confirming that the nanocomposite had a high synergistic therapeutic effect [49], and its photothermal properties promoted the improvement in peroxidase activity under NIR laser irradiation. Meanwhile, autophagy induced more cell death.

Also, the killing capability of MXene@PDA/Co-N-CDs was further evaluated using the Annexin V-FITC/PI double staining method, and the apoptosis status of tumor cells after different treatments was analyzed by flow cytometry (Figure S3). The results indicated that the control group cells exhibited an overall good condition, mostly existing in the Q1-LL region (96.11%, Annexin V-FITC-/PI-). The laser-treated cells displayed a similar overall condition to the control group (95.75%, Annexin V-FITC-/PI-). In contrast, MCF-7 cells treated with MXene@PDA/Co-N-CDs were predominantly located in the Q1-LL region (68.02%, Annexin V-FITC+/PI-), while MCF-7 cells treated with MXene@PDA/Co-N-CDs + laser light were mostly found in the Q1-UR region (41.79%, Annexin V-FITC+/PI+), indicating late apoptosis, which was significantly different from the control group.

Using in vitro experiments, by observing and measuring the response and changes in MCF-7 cells under different treatment conditions, it is possible to evaluate the effect of the synergistic therapeutic effect of PTT and photothermal enhanced POD (peroxidase) catalytic therapy on cells. The results showed that the proliferation of MCF-7 cells was significantly inhibited, and the cell viability was significantly reduced, indicating that the material had a synergistic effect between MXene and Co-N-CDs for enhancing the therapeutic effect.

## 4. Conclusions

In summary, we fabricated a novel MXene@PDA/Co-N-CDs nanozyme with high enzyme-mimic activity and excellent photothermal capability by depositing Co-N-CDs onto MXene@PDA nanosheets. The well-encapsulated PDA improved the biocompatibility and hydrophilicity of MXene, while Co-N-CDs endowed the nanozyme with outstanding catalytic properties. By combining MXene with PDA and Co-N-CDs, the POD catalytic activity was remarkably improved. Based on MXene@PDA/Co-N-CDs, a highly sensitive and selective colorimetric detection method for H_2_O_2_ and GSH was established, which showed significant potential in biomedical applications. In the practical analysis of human urine and serum samples, the application of MXene@PDA/Co-N-CDs obtained a satisfactory recovery rate. In addition, MXene@PDA/Co-N-CDs as activated CDT agents could catalyze the hydrogen peroxide overexpressed in cancer cells to produce excessive ROS and induce oxidative stress to achieve tumor catalytic therapy. Meanwhile, the effective ablation of tumors could be achieved by hyperthermia-enhanced nanozyme catalytic therapy through NIR-I laser irradiation. This study provided a novel nanozyme with POD-like activity, which not only enabled the colorimetric detection of GSH but also enhanced the ability of tumor kinetic therapy in combination with its superior photothermal effect. MXene@PDA /Co-N-CDs are an excellent candidate material for nanocatalysts and have broad application prospects in biosensing, medical diagnosis, and disease treatment.

## Figures and Tables

**Table 1:** Comparisons of the kinetic behavior of different nanozymes.

Catalytic Material	Km (mM)		Vm (10^−8^ M·s^−1^)		Reference
	TMB	H_2_O_2_	TMB	H_2_O_2_	
HPR	0.434	3.7	10	8.71	[37]
K,Cl-L-CDs	0.85	0.19	11.63	251	[23]
MXene@NiFe-LDH	0.187	0.078	1.707	2.076	[38]
GOx@ZIF-8@Fe-PDA	0.21	0.09	0.74	0.30	[39]
N/S-CDs	0.0765	0.0488	0.3096	0.6799	[40]
MXene@PDA/Co-N-CDs	0.017	0.6949	0.0486	0.3437	This work

**Table 2:** Comparison of the MXene@PDA/Co-N-CDs colorimetric sensing assay with the reported colorimetric methods for GSH detection.

Probe	Linear Range (μM)	LOD (μM)	References
NS-CQDs	1–55 μM	4.0 μM	[41]
CDs@ZIF-8	0–100 μM	1.04 μM	[42]
Co-POP	5–300 μM	0.71 μM	[43]
MXene@PDA/Co-N-CDs	0.3–20 μM	0.12 μM	this work
Si-CoO	1–100 μM	0.45 μM	[44]
Pt/ZnFe_2_O_4_/C nanoperoxidase	1–30 μM	0.38 μM	[45]
Graphene Dots	0.5–100 μM	0.5 μM	[46]
TiO_2_/C-QDs	0–25 μM	0.2 μM	[47]

**Table 3:** Detection of GSH in 10-fold dilution of urine samples (*n* = 3).

Urine	Addition (µM)	Found (µM)	Recovery (%)	RSD (%)
Urine 1	0	0.05	-	4.6
	2.5	2.45	98	5.1
	3	3.03	101	3.2
	4	4.04	101	2.6
Urine 2	0	0.02	-	4.7
	2.5	2.41	96	3.6
	3	3.13	110	4.4
	4	4.03	101	4.5
Urine 3	0	0.08	-	3.8
	2.5	2.51	101	4.6
	3	3.14	105	5.1
	4	3.86	97	3.2

**Table 4:** Detection of GSH in 50-fold dilution of serum samples (*n* = 3).

Serum	Found (µM)	Addition (µM)	Found (µM)	Recovery (%)	RSD (%)
Serum 1	0.178	5	5.36	104	1.48
		10	9.91	97	4.34
		20	19.84	98	2.64
Serum 2	0.179	5	5.26	102	0.44
		10	10.54	104	0.92
		20	20.74	103	2.07
Serum 3	0.25	5	5.68	109	0.86
		10	10.56	103	0.92
		20	20.73	102	0.46

## Data Availability

Data is contained within the article.

## Supplementary Materials

The following supporting information can be downloaded at https://www.mdpi.com/article/10.3390/nano14131090/s1: Figure S1: AFM graphs of MXene (**a**,**b**) and the zeta potentials of the different samples (**c**); Figure S2: The peroxidase-like catalytic activity of MXene@PDA/Co-N-CDs with TMB (**a**) as a substrate were affected by temperature (**b**) and pH (**c**); Figure S3: The percentage of apoptotic cells measured by flow cytometry using an Annexin-V-FITC/PI apoptosis detection kit.
