# Supplementary material for: MXene/Carbon Dots Nanozyme Composites for Glutathione Detection and Tumor Therapy
Source: Nanomaterials (Basel). 2024 Jun 25;14(13):1090. doi: 10.3390/nano14131090 (PMC11243324; doi:10.3390/nano14131090)
Supplement: Supplementary file 1 [file nanomaterials-14-01090-s001.zip › nanomaterials-3025778-supplementary.pdf]

Supporting Information

MXene/Carbon Dots Nanozyme Composites for Glutathione Detection and Tumor Therapy

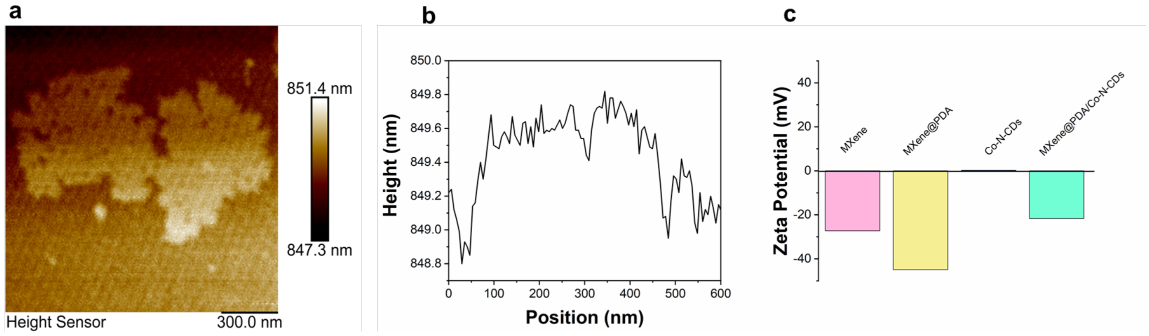

Figure S1. AFM graphs of MXene (a, b) and the zeta potentials of the different samples (c).

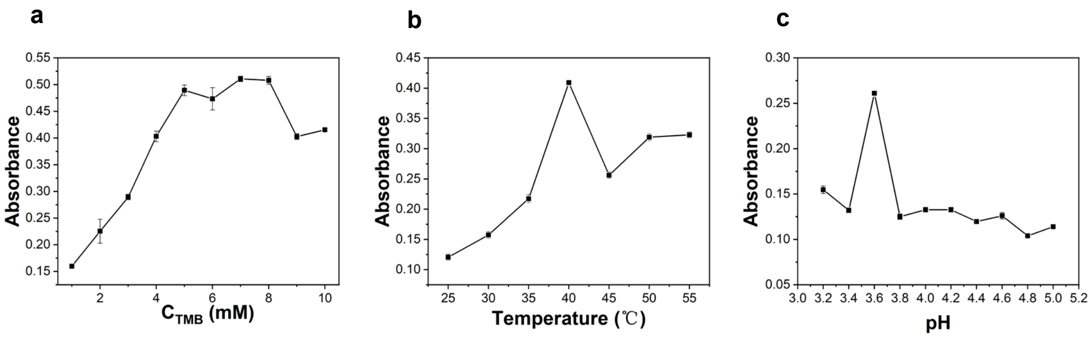

Figure S2. The peroxidase-like catalytic activity of MXene@PDA/Co-N-CDs with TMB (a) as substrate were affected by temperature (b) and pH (c).

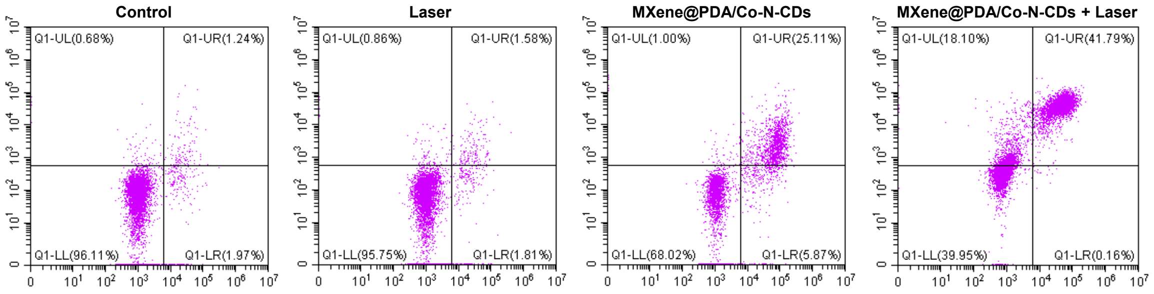

Figure S3. The percentage of apoptotic cells measured by flow cytometry using Annexin-V-FITC/PI apoptosis detection kit.
